# Colorimetric Humidity Sensor Using Inverse Opal Photonic Gel in Hydrophilic Ionic Liquid

**DOI:** 10.3390/s18051357

**Published:** 2018-04-27

**Authors:** Seulki Kim, Sung Gu Han, Young Gook Koh, Hyunjung Lee, Wonmok Lee

**Affiliations:** 1 Department of Chemistry, Sejong University, 209 Neungdong-ro, Gwngjin-gu, Seoul 05006, Korea; 01086206199@naver.com (S.K.); chemiboy85@gmail.com (S.G.H.); 2 Engain Co. Ltd. Korea Bio Park BLD C-201, Seongnam 13488, Korea; young.koh@engain.co.kr; 3 School of Advanced Materials Engineering, Kookmin University, 77 Jeongneung-ro, Seongbuk-gu, Seoul 02707, Korea; hyunjung@kookmin.ac.kr

**Keywords:** colorimetric sensor, humidity sensor, inverse opal photonic gel, hydrophilic ionic liquid

## Abstract

We demonstrate a fast response colorimetric humidity sensor using a crosslinked poly(2-hydroxyethyl methacrylate) (PHEMA) in the form of inverse opal photonic gel (IOPG) soaked in 1-butyl-3-methylimidazolium tetrafluoroborate ([BMIM^+^][BF_4_^−^]), a non-volatile hydrophilic room temperature ionic liquid (IL). An evaporative colloidal assembly enabled the fabrication of highly crystalline opal template, and a subsequent photopolymerization of PHEMA followed by solvent-etching and final soaking in IL produced a humidity-responsive IOPG showing highly reflective structural color by Bragg diffraction. Three IOPG sensors with different crosslinking density were fabricated on a single chip, where a lightly crosslinked IOPG exhibited the color change response over entire visible spectrum with respect to the humidity changes from 0 to 80% RH. As the water content increased in IL, thermodynamic interactions between PHEMA and [BMIM^+^][BF_4_^−^] became more favorable, to show a red-shifted structural color owing to a longitudinal swelling of IOPG. Highly porous IO structure enabled fast humidity-sensing kinetics with the response times of ~1 min for both swelling and deswelling. Temperature-dependent swelling of PHEMA in [BMIM^+^][BF_4_^−^] revealed that the current system follows an upper critical solution temperature (UCST) behavior with the diffraction wavelength change as small as 1% at the temperature changes from 10 °C to 30 °C.

## 1. Introduction

There has been growing interest in the utilization of opal templating method for the fabrication of various photonic crystal (PC) devices such as stimuli-responsive colorimetric sensors and reflective full color displays [1,2]. As a nature-mimicking process, opal templating provides manifold advantages compared to other fabrication methods, such as simple and low-cost process, color tunability, various external stimuli, etc. [3,4]. The origin of colors in PC stems from its periodic structure with a lattice spacing d which reflects incident light of a specific wavelength *λ* at an angle of diffraction *θ* as represented by Equation (1), and thus the reflected color is often called as the structural color.

2*n*_eff_*·d*·sin *θ* = *λ* (1)

If *d* or the effective index of refraction (*n*_eff_) can be varied by certain stimulus, the structural color will be changed as well, and such a PC will act as a stimuli-responsive colorimetric sensor. A light diffraction can occur either longitudinally or laterally. A longitudinal light diffraction is utilized in one-dimensional (1D) PC (e.g., multi-layered structure, opal film with {111} facet parallel to the substrate) to which Bragg equation can be applied, while a lateral diffraction occurs in two-dimensional (2D) PC such as diffraction grating or colloidal monolayer. To be utilized as a colorimetric sensor, likewise, d should be changed longitudinally in 1D-PC, while a lateral variation of d should occur in 2D-PC. Comprehensive studies have been conducted towards the fabrications of stimuli-responsive sensors using 1D- or 2D-PC structures via opal templating techniques. From the mechanistic point of view, PC sensors can be either field-responsive (e.g., electric field [5,6,7,8], magnetic field [9], pressure [10], temperature [11,12,13]) or mass-responsive, and the latter is often called as a chemical sensor. Various examples of analytes can be investigated in chemical sensor such as ions in aqueous phase, and gaseous species as well. For fabrication of PC sensors, volume-changeable hydrogels are generally utilized [14,15,16]. In a variety of PC chemical sensors, the driving force of hydrogel swelling is related to the development of osmotic pressure occurring at the interface between the hydrogel and the bulk solution, which is induced by distinct distribution of ionic charges between them. On the other hand, there also have been studies on the PC chemical sensors based on thermodynamically driven swelling/deswelling of hydrogel in aqueous phase upon inclusion of the gaseous analytes (e.g., water vapor, ammonia, CO_2_) [17,18,19,20]. T. Kanai et al. reported a swelling of gel-immobilized colloidal PC in a hydrophilic ionic liquid (IL) [21]. Smith et al. reported an opal-templated 2D PC gas sensor soaked in 1,3-diallylimidazolium bis(trifluoromethanesulfonyl)imide IL which showed a color change responses to water or ammonia vapor [20]. Tian et al. reported that an opal templated copolymers of styrene, methylmethacrylate, and acrylamide can be used as a PC humidity sensor [18]. In the aforementioned studies on humidity sensors, however, none of them utilized a porous structure which can provide a rapid response kinetics. Recently, Barry et al. reported a humidity sensor having a porous inverse opal structure of polyacrylamide which showed a rapid response time (~20 s) for humidity sensing, while the sensitivity was relatively poor [17]. In general, a fast response kinetics for humidity sensing can be achieved by incorporating an intermediate liquid phase like IL for water sorption [18]. In this study, we investigate a crosslinked inverse opal photonic gel (IOPG), which is soaked in 1-butyl-3-methylimidazolium tetrafluoroborate ([BMIM^+^][BF_4_^−^]), a non-volatile hydrophilic room temperature ionic liquid (IL) to demonstrate a fast-response and high-sensitivity humidity sensor.

## 2. Materials and Methods

Materials: Styrene (98%), potassium persulfate, sodium dodecylsulfate (SDS), activated alumina, trichlorooctadecyl silane, ethylene glycol dimethacrylate (EGDM) (98%), and iso-octane were purchased from Sigma-Aldrich, (St. Louis, MO, USA). 2-hydroxyethyl methacrylate (HEMA) (96%) was supplied from Junsei, (Tokyo, Japan) and Irgacure-651 was obtained from Ciba specialty chemicals, (Basel, Switzerland). Ionic liquid, [BMIM^+^][BF_4_^−^] was supplied by Future Chem, (Seoul, Korea). Chloroform and acetonitrile (ACN) was respectively purchased from Duksan Chemical (Seoul, Korea) and Daejung Inc. (Seoul, Korea). Deionized (DI) water was produced from the water purifying system (human technology).

**Figure 1:**
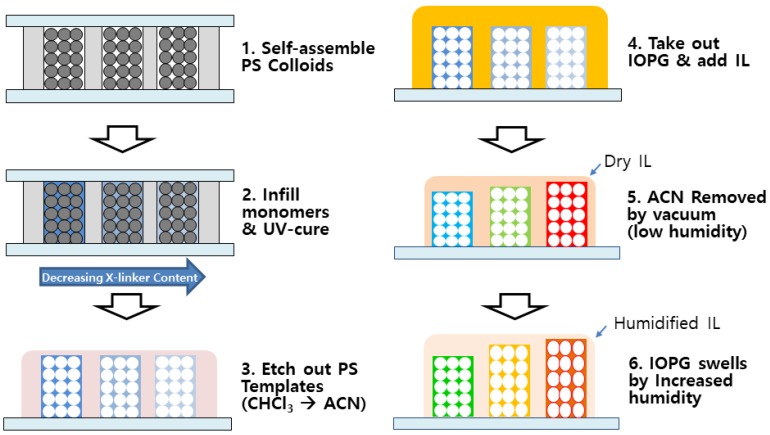
Schematic illustration showing fabrication procedures of PHEMA IOPG, and its swelling behavior under humidified condition.

Methods: The polystyrene (PS) μ-sphere with narrow size distribution was synthesized by emulsion polymerization [22]. In an N_2_-purged DI water which is heated at 70 °C, potassium persulfate and SDS were fully dissolved, and emulsion polymerization was carried out upon addition of styrene which had been filtered through activated alumina for inhibitor removal. After polymerization for 4 h, the μ-spheres dispersion was filtered through pre-cleaned cotton fibers, and dialyzed using a semi-permeable cellulose membrane tube (MWCO 12,000–14,000, MFPI) for removal of the remaining impurities. The purified aqueous dispersion of μ-sphere was about 15 wt %, and the average diameter was characterized to be 220 nm. For fabrication of IOPG humidity sensor, a recently developed technique, called a ‘directed enhanced evaporation of water for colloidal assembly’ (DEECA), was used, as schematically shown in Figure 1 [23]. The template was composed of three parts, a top slide, a spacer, and a bottom slide. To make a top slide, a standard size glass slide (2 × 6 cm^2^) with three drilled holes (diameter = 1 mm) was treated with trichlorooctadecyl silane (Sigma) in *i*-octane to render it hydrophobic. As a spacer, 30 μm-thick Surlyn^®^ (Dupont, Wilmington, DE, USA) film was cut to form three individual channels when hot-pressed between a top and a bottom slide. Upon completion of assembly, the bottom of each channel was wide open, while the drilled hole was placed at the top of each channel. An aliquot of aqueous suspension of 15 wt % PS μ-sphere was infiltrated from the bottom of each channel, and the top holes were sealed with Teflon^®^-tape (Dupont). After 4 h of colloidal assembly induced by water evaporation, the DEECA cell was further air-dried for 24 h, and thermally annealed at 80 °C for 3 h. A precursor mixture of 2.5 g of 2-hydroxyethyl methacrylate (HEMA) (96% Junsei), 0.025 g of ethylene glycol dimethacrylate (EGDM) (98%, Sigma-Aldrich), 0.075 g of Irgacure-651^®^ (Ciba specialty chemicals), and 0.625 g of DI water was prepared, and a small aliquot of precursor mixture was infiltrated within the interstices of the colloidal assembly in DEECA cell, which was photopolymerized by exposure to a UV lamp (Spectroline, MODEL5B-100P/F) for 1 h. After removing the top slide from the DEECA cell, PS μ-spheres were etched away by immersing the cell in chloroform bath for one day, and the resulting IOPG was subsequently rinsed with chloroform and ACN. The IOPG in ACN was transferred to [BMIM^+^][BF_4_^−^] IL, and finally vacuum-dried at 40 °C overnight in order to completely remove ACN leaving the dried IL behind. An as-prepared IOPG in IL was placed in a custom-made desiccator (LK Lab Korea) in which the relative humidity had been kept very low (<1.0% RH) by using CaSO_4_ drierite. The relative humidity was monitored by using a digital humidimeter (Traceable^®^, Control Company, Webster, NY, USA). The humidity was increased by placing wet cotton balls in the desiccator. The color changes of IOPG were characterized by a digital camera or a custom-made reflectance measurement system equipped with a reflective microscope (L2003A, Bimeince, Seoul, Korea) and a UV–vis spectrometer (AvaSpec^®^, Avantes, Apeldoorn, The Netherland). Refractive index of IL with varying water content was measured using a digital refractometer (RX-5000a, Atago, Tokyo, Japan).

## 3. Results and Discussion

**Figure 2:**
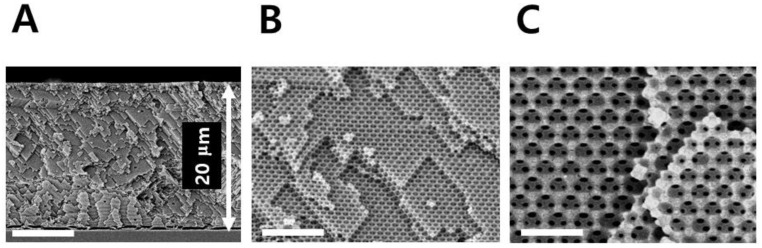
Scanning electron microscopic images showing the cross-sections of inverse opal structure of cross-linked PHEMA IOPG used in this study. Scale bars indicate 7 (**A**), 3 (**B**), and 1 μm (**C**) respectively.

The sensor material used in this study is a cross-linked PHEMA which is well known for hydrophilicity, biocompatibility, and rubber elasticity [24]. Opal-templated photopolymerization of PHEMA via DEECA process provided a 20 μm-thick IOPG film as shown in Figure 2.

It was reported that polyvinylalcohol (PVA), a typical water-soluble polymer is compatible with a hydrophilic IL of [BMIM^+^][BF_4_^−^], while the thermodynamic interaction between PVA and IL is controlled by inclusion of water in IL [25,26]. We found that PHEMA is also compatible with [BMIM^+^][BF_4_^−^], and an IOPG film of PHEMA in [BMIM^+^][BF_4_^−^] was found to exhibit a humidity-dependent color changing response. At a low humidity, IL molecules are equilibrated with PHEMA IOPG. Upon the increase of humidity, water is dissolved in IL since BF_4_^−^, a kosmotropic ion strongly interacts with water molecule by hydrogen bonding [26]. Following this, the osmotic pressure is developed within IOPG, and there are mutual diffusions of water and IL molecules in and out of IOPG, where lighter water molecules can diffuse into IOPG faster than IL to bring about rapid longitudinal swelling of IOPG, and consequently red-shift the diffraction wavelength in Equation (1). In the meantime, a hydrophobic BMIM^+^ behave as a chaotropic ion which disrupts the hydrogen bonding between water and PHEMA to exclude water molecules out of IOPG during the drying process, so the shrinking of IOPG and the blue-shift of the structural color occurs. In some circumstances, BMIM^+^ is also reported to act as a kosmotropic ion due to hydrophobic hydration [26].

In addition to the swelling and deswelling of IOPG to induce color change of humidity sensor, *n*_eff_ of IOPG and liquid medium can also affect the structural color as Equation (1) states. In Table 1, the refractive index (*n*_eff,D_; effective refractive index of IL/H_2_O mixture measured with Na D-line) values of [BMIM^+^][BF_4_^−^] under various humidities at 25 °C are shown. *n*_D_ of a dried [BMIM^+^][BF_4_^−^] IL was measured to be 1.4230, and that of DI water was 1.3330. Even though *n*_eff,D_ showed a decreasing tendency with an increased humidity due to inclusion of water with low index of refraction, the values were maintained close to that of pure IL regardless of relative humidity, implying that apparent water content in IL is kept low. Using Equation (2), water content (*f*_water_) was calculated for the measured *n*_eff,D_ values (Table 1, and Supplementary Material, Figure S1).

*n*_eff,D_ = *n*_water_ × *f*_water_ + *n*_IL_ × (1 − *f*_water_) (2)

**Figure 3:**
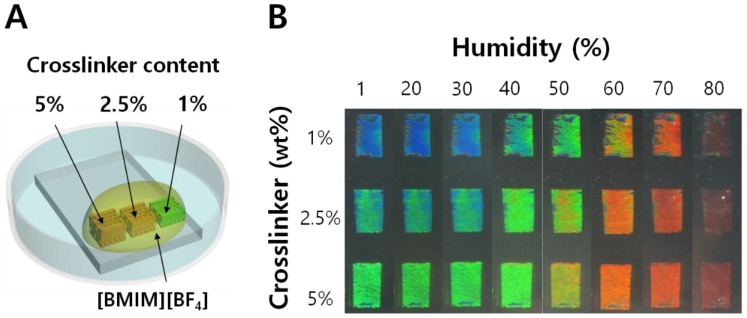
(**A**) Illustration showing three IOPGs with different crosslinker contents on a single substrate which are soaked in [BMIM^+^][BF_4_^−^] IL; (**B**) Humidity-dependent color changes of three PHEMA IOPGs with crosslinker contents of 1, 2.5, and 5%. Upon variations of relative humidity from 1% to 80% RH, a less-crosslinked (1% EGDM content) IOPG shows entire visible color ranges.

The calculated *f*_water_ tells us about the limited solubility of water in [BMIM^+^][BF_4_^−^] even under a highly humid condition. From the insensitive *n*_eff,D_ values at different humidities, we can ignore its contribution to the structural color, and can interpret the color changes only with the longitudinal swelling of IOPG during humidity variation. The evaporative loss of [BMIM^+^][BF_4_^−^] can also be ignored due to its extremely low vapor pressure. In the fabrication of humidity sensors, the precursor mixtures of three different crosslinker contents (1, 2.5, and 5%) with respect to HEMA were respectively prepared, and the IOPGs with different crosslinking densities were fabricated on a single glass substrate as shown in Figure 3A.

Entire IOPGs were fully soaked with [BMIM^+^][BF_4_^−^], open to air. The IOPG/IL humidity sensors were subject to the controlled humidity, to reveal the color changes as shown in Figure 3B. The temperature for each humidity condition was maintained at 25 ± 1 °C in order to exclude a possible temperature-driven swelling of IOPG, which will be discussed later in this paper. Figure 3B clearly demonstrates that the increase in crosslinking density of the IOPGs resulted in a red-shifted structural color response. For instance, the structural color of 1% crosslinker-containing IOPG was blue at a low humidity condition while that of 5% crosslinker exhibits a green color. The fewer the crosslinks, the wider color spectra were obtained for IOPG/IL sensor. By fabricating several batches of the humidity sensors, and observing the responses individually, the reproducibility for sensor production was confirmed to be good enough (Supplementary Material, Figure S2).

**Figure 4:**
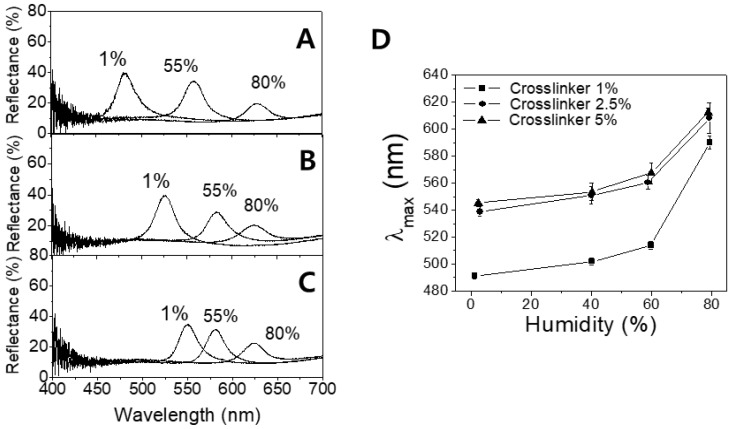
Reflectance spectra of PHEMA IOPGs with different crosslinker contents of (**A**) 1%; (**B**) 2.5%; (**C**) 5%; and (**D**) plot of *λ*_max_ vs. humidity.

In order to investigate the sensor responses in more quantitative manner, the reflectance spectra for the respective IOPG/IL sensors were obtained. In Figure 4A–C, the spectra at three different humidity values are shown where Figure 4A shows the data from a sensor containing 1% crosslinker, and (b) and (c) are from 2.5% and 5% crosslinker-containing sensors respectively.

**Figure 5:**
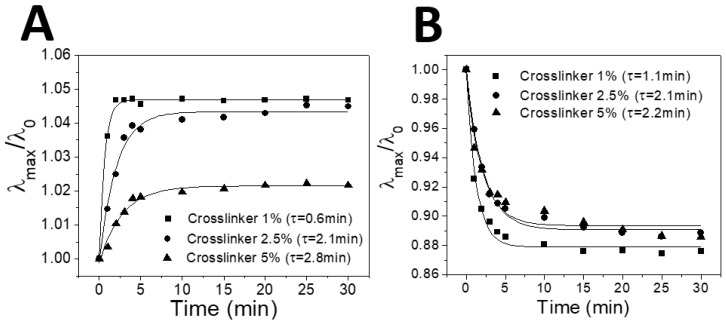
Response kinetics for (**A**) swelling (humidity changes from 1% to 40% RH) and (**B**) deswelling (humidity changes from 80% to 40% RH) of three IOPGs with different crosslinking densities. Solid curves are the exponential fitting to the respective plots, and the obtained time constants (*τ*) are shown. The IOPG with the lowest crosslinking density (1 wt %) shows the most rapid swelling and deswelling kinetics.

It is obvious from Figure 4A that a less-crosslinked IOPG sensor shows better-resolved peaks at the given humidity (1%, 55%, and 80% RH) just like the color responses demonstrated in Figure 3B. Such tendencies imply that a more-crosslinked IOPG is stronger in mechanical strength, and less stretchable. It is a general trend yet worthwhile to note that in all three sensors, the reflectance peaks get weaker as humidity increases, since a more-swollen IOPG exhibits a smaller Δ*n* between IOPG and liquid medium which is the origin of light diffraction. The peak wavelengths (*λ*_max_) in the respective spectra were plotted with respect to the humidity values as shown in Figure 4D, in which the red-shifts are evident for IOPGs with higher crosslinker contents. The repeated variations of low and high humidities have revealed that there is no hysteresis of *λ*_max_, and the reproducibility was as good as shown by error bars in Figure 4D. For individual measurements of humidity-dependent color changing responses, each humidity condition was maintained for at least 20 min prior to the reflectance measurement, so that the distribution of water molecules reached the equilibrium within the IOPG/IL. To confirm whether 20 min is enough duration for the equilibrium, a humidity-dependent swelling/deswelling kinetics of IOPG/IL was investigated. In Figure 5, the plots of relative wavelength ratios of time-dependent *λ*_max_ with respect to the initial *λ*_max_ (*λ*_0_ at *t* = 0) are shown upon instantaneous humidity changes. Figure 5A shows the kinetics plots for swelling, in which differently crosslinked IOPGs which had been stored at a low humidity (1% RH) were suddenly exposed to air with 40% RH. In Figure 5B, the same IOPGs at a high humidity (80% RH) were taken out to an environment with 40% RH.

**Figure 6:**
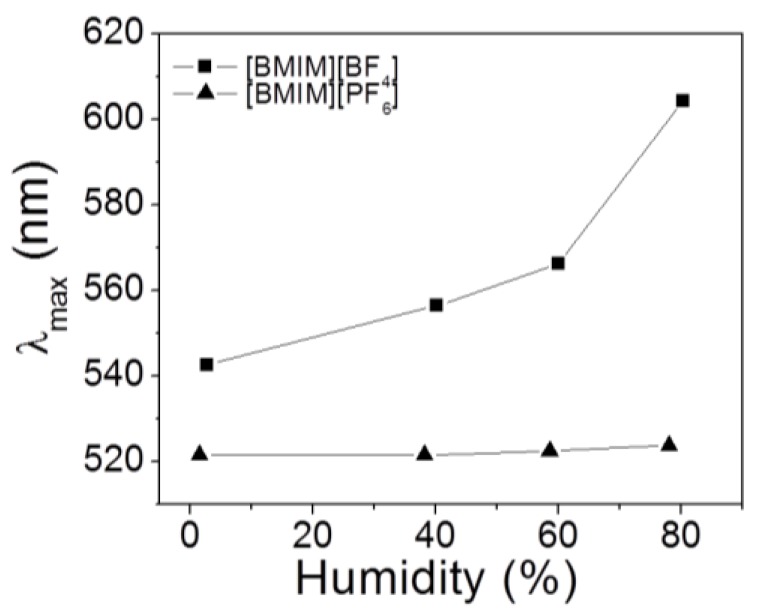
Comparison of wavelength shifts for 2.5%-crosslinked PHEMA IOPG soaked in two different ILs of [BMIM^+^][BF_4_^−^] and [BMIM^+^][PF_6_^−^] with respect to humidity change.

During entire experiments, the temperature was maintained at 25 ± 1 °C. The respective data were fitted to the single exponential functions, as the fitted curves and time constants (*τ*) are shown in Figure 5. A lightly-crosslinked IOPG (with 1% crosslinker) showed the fastest *τ* of 0.6 min and 1.1 min respectively for swelling and deswelling processes due to larger free volumes within the IOPG, while a highly-crosslinked (5%) IOPG showed 2–3 times longer *τ* of 2.8 min and 2.2 min, respectively (please refer to a movie clip showing a deswelling of the same IOPGs used in Figure 5). However, all of the time constants were less than 3 min which are much faster than the equilibration time of 20 min for the humidity sensing experiments. A rapid humidity sensing shown in Figure 5 could be achieved owing to highly porous structures of IOPGs used in this study as shown in Figure 1, as well as a rapid diffusion of water vapor into IL [13,27]. Despite PHEMA IOPGs in [BMIM^+^][BF_4_^−^] showed Full RGB color responses due to a decent longitudinal swelling within humidity ranges of 0% to 80% RH, not every IL can be employed for this purpose. A less hydrophilic IL, 1-Butyl-3-methylimidazolium hexafluorophosphate ([BMIM^+^][PF_6_^−^]) did not show noticeable color changing responses under the given humidity variations as shown in Figure 6.

**Figure 7:**
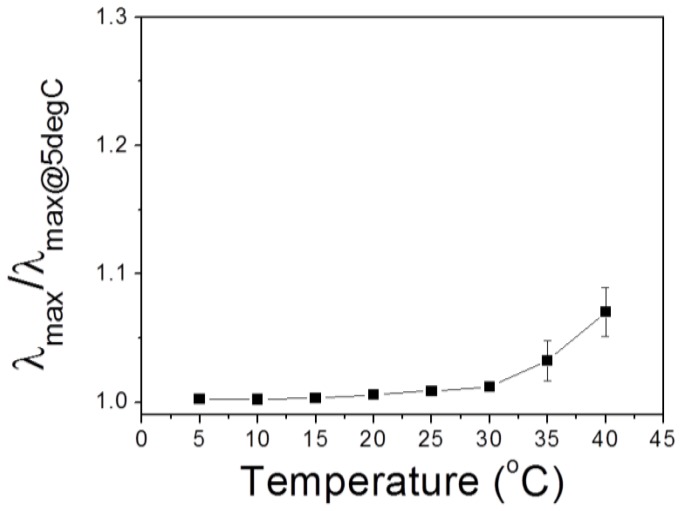
Temperature dependence on IOPG swelling. 2.5%-crosslinked PHEMA gel was used. Humidity was maintained at 20 ± 1% RH.

Individual spectra for Figure 6 with varying humidity are shown in Supplementary Material, Figure S3. Inappreciable color changes of PHEMA IOPG in [BMIM^+^][PF_6_^−^] implies that its water solubility is negligible in that IL, and thus no significant energetic change occurs between IL and IOPG to induce a gel swelling. Due to existence of fluorine atoms, PF_6_^−^ anion makes the imidazolium salt much similar to organic solvents and less soluble in water as well [28]. Although the experiments were carried out under the thermostated conditions, a temperature dependence of IOPG in [BMIM^+^][BF_4_^−^] under a fixed humidity was examined. From a thermodynamic point of view, temperature increase will induce swelling of PHEMA IOPG assuming that HEMA and IL/water has upper critical solution temperature (UCST) behavior. As shown in Figure 7, temperature increase resulted in a redshift of *λ*_max_ as expected. However, the degree of wavelength change (*λ*_max_/*λ*_max @ 5°C_), which is an indication of longitudinal IOPG swelling, was less than 1% at the temperature ranges lower than 30 °C. Therefore, the reliability of the humidity sensor can be guaranteed within the temperature ranges of 10 °C to 30 °C.

## 4. Conclusions

In summary, we fabricated a crosslinked IOPG via DEECA followed by photopolymerization of HEMA and crosslinker which was subsequently soaked in [BMIM^+^][BF_4_^−^], a non-volatile hydrophilic IL at room temperature to demonstrate a colorimetric humidity sensor. Under varying humidity from 0% to 80% RH, IOPGs with different crosslinker contents were exposed, to exhibit structural color changes over entire visible ranges especially for a lightly crosslinked IOPG. Fast color change responses with exponential time constants of 0.6~2.8 min were obtained due to highly porous IO structure of the sensor. From a temperature-dependent color change test, temperature increase resulted in a swelling of IOPG, implying that the thermodynamic interaction between PHEMA IOPG and [BMIM^+^][BF_4_^−^] is supposedly UCST behavior. However, the dimensional change was less than 1% at the temperature variations from 10 °C to 30 °C.

## Figures and Tables

**Table 1:** Water content in IL at various humidity calculated from refractive index.

Relative Humidity (% RH)	0	1	38	58	76
Refractive index (*n*D)	1.4230	1.4224	1.4215	1.4201	1.4159
Water content in IL (%)	0	0.5	1.5	2.6	7.3

Note: All values were obtained at 25 °C.

## Supplementary Materials

The following are available online at http://www.mdpi.com/1424-8220/18/5/1357/s1, Figure S1: Plot of water fraction vs. humidity. Water fraction was calculated by the following equation; Figure S2: (a) Two of PHEMA (with crosslinker 2.5%) IOPGs on a single substrate having the same chemical compositions soaked in [BMIM][BF4] (b) Cross-section SEM Images of the IOPG; Figure S3: (a) Reflectance spectra of PHEMA(with crosslinker 2.5%) IOPG in ionic liquid of 1-Butyl-3-methylimidazolium hexafluorophosphate ([BMIM][PF6]) showing no wavelength shift at different humidity (b) Molecular structure of [BMIM][PF_6_] ionic liquid.
