# Supplementary figures and images for: Colorimetric Humidity Sensor Using Inverse Opal Photonic Gel in Hydrophilic Ionic Liquid
Source: Sensors (Basel). 2018 Apr 27;18(5):1357. doi: 10.3390/s18051357 (PMC5982702; doi:10.3390/s18051357)

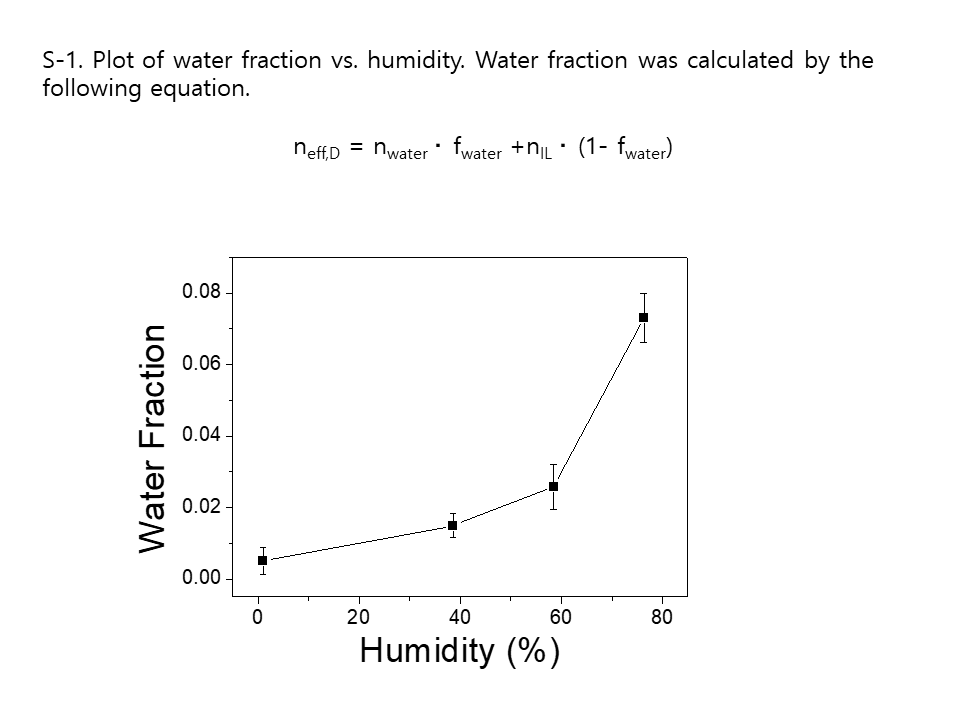

Supplement: Supplementary file 1 [file sensors-18-01357-s001.zip › Supplementary material-Sensors/S-1.TIF]

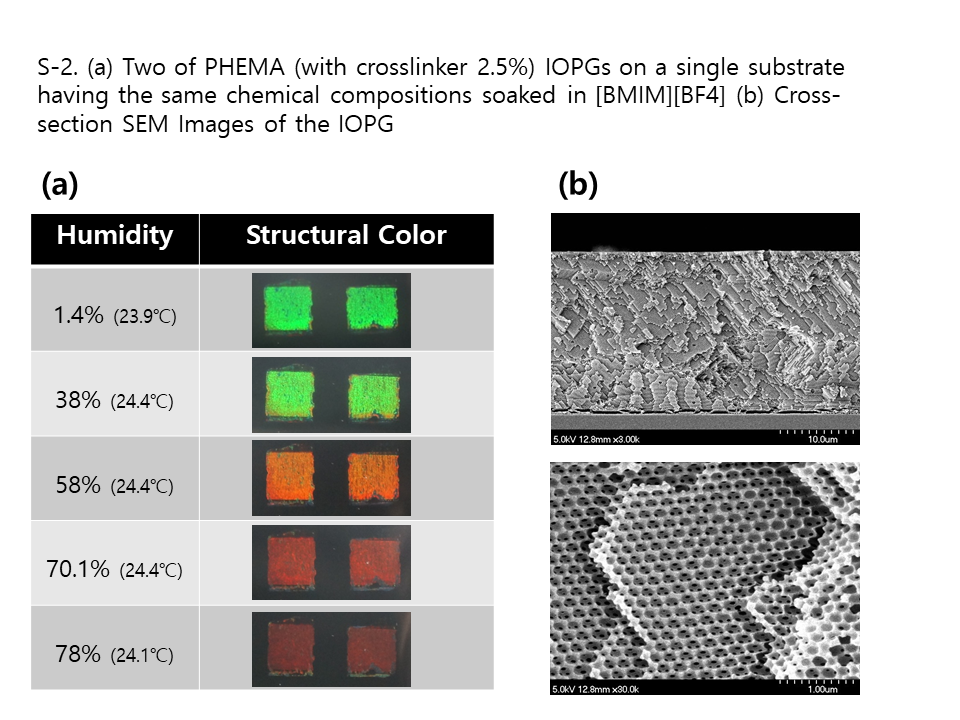

Supplement: Supplementary file 1 [file sensors-18-01357-s001.zip › Supplementary material-Sensors/S-2.TIF]

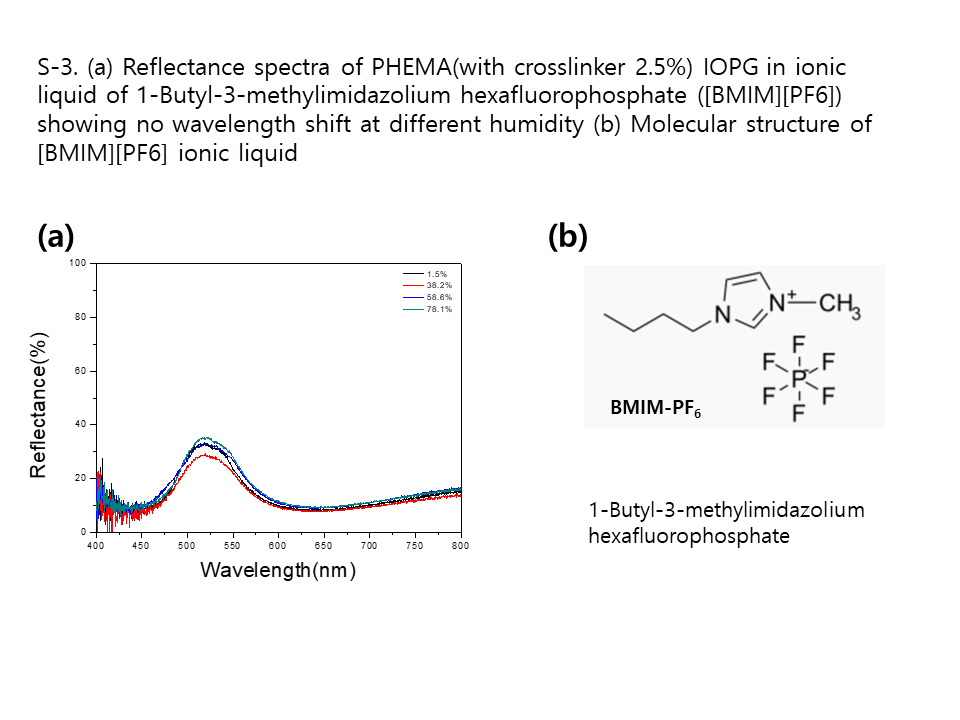

Supplement: Supplementary file 1 [file sensors-18-01357-s001.zip › Supplementary material-Sensors/S-3.TIF]
